# The impact of supplementary immunization activities on routine vaccination coverage: An instrumental variable analysis in five low-income countries

**DOI:** 10.1371/journal.pone.0212049

**Published:** 2019-02-14

**Authors:** Averi Chakrabarti, Karen A. Grépin, Stéphane Helleringer

**Affiliations:** 1 Department of Public Policy, The University of North Carolina at Chapel Hill, Chapel Hill, NC, United States of America; 2 Department of Health Sciences, Wilfrid Laurier University, Waterloo, ON, Canada; 3 Department of Population, Family and Reproductive Health, Bloomberg School of Public Health, Johns Hopkins University, Baltimore, Maryland, United States of America; ESIC Medical College & PGIMSR, INDIA

## Abstract

**Background:**

Countries deliver vaccines either through routine health services or supplementary immunization activities (SIAs), usually community-based or door-to-door immunization campaigns. While SIAs have been successful at increasing coverage of vaccines in low- and middle-income countries, they may disrupt the delivery of routine health services. We examine the impact of SIAs on routine vaccine coverage in five low-income countries.

**Methods:**

Data on the number and timing of SIAs conducted in various countries was compiled by WHO and obtained through UNICEF. Information on the coverage of vaccines not targeted by SIAs (e.g., DPT) was extracted from the Demographic and Health Surveys. We focus on SIAs that took place between 1996 and 2013 in Bangladesh, Senegal, Togo, Gambia, and Cote d’Ivoire, and examine outcomes for children aged 12–59 months. To avoid biases resulting from non-random placement and timing of SIAs, we use age of a child at her first SIA as an instrumental variable for total exposure to SIAs.

**Results:**

We find that SIA exposure reduced the likelihood of receiving routine vaccines in all the countries included in the study; the coefficients of interest are however statistically insignificant for Gambia and Cote d’Ivoire. In countries that witnessed statistically significant SIA-induced declines in the likelihood of obtaining DPT 3, measles as well as BCG, reductions ranged from 1.3 percentage points (Senegal) to 5.5 percentage points (Bangladesh).

**Conclusion:**

SIA exposure reduced routine vaccination rates in study countries. Efforts should be made to limit the detrimental impact of SIAs on the services provided by routine health systems.

## Introduction

Countries deliver vaccines either through routine health systems or through supplementary immunization activities (SIAs). SIAs are immunization campaigns which have been used to rapidly scale-up coverage of key immunizations. They are primarily used for the distribution of polio and measles vaccines. Since 1988, these campaigns have been widely implemented around the world [1]. SIAs have brought about a substantial reduction in polio cases and successfully contained wild polio virus transmission to only a few countries [2]. SIAs are usually implemented by national governments with the assistance of international organizations such as the World Health Organization (WHO) and the United Nations Children’s Fund (UNICEF) [3]. The types of SIAs vary—they are carried out country-wide during national immunization days (NIDs), in regions with a high risk of polio transmission during sub-national immunization days (SNIDs), as part of child health days (CHDs) that provide maternal and child healthcare, or through mop-up rounds that aim to stem any chains of polio transmission that remain in a region [4,5].

While SIAs have been effective at increasing coverage of key vaccines and achieving quick results, they might unintentionally disrupt routine health service delivery [1,6,7]. One examination of polio eradication efforts in several countries indicated that polio campaigns enabled widespread distribution of vitamin A supplements and linked health workers to communities, among other benefits [8]. In contrast, an SIA campaign in South Africa was found to be associated with a reduction in child and maternal health services [9]. Another analysis suggested that NIDs contributed to the effectiveness of routine immunization in India, Nepal, Cote d’Ivoire, and Ghana, but had the opposite effect in Nigeria and Zimbabwe [10]. Vaccine campaign exposure had negligible consequences for routine vaccination coverage in rural North India, reduced routine services in Cameroon, and improved the utilization of routine immunization services in Bangladesh [11–13]. A multi-country analysis found that in some contexts, there were benefits such as improved disease surveillance, whereas in areas with frequent campaigns, there was disruption of services [14]. An analysis of the polio eradication initiative in two Indian states found positive effects on routine immunization performance in Bihar, but negative effects in Uttar Pradesh [15]. A recent review article summarizes the evidence from several studies examining the impacts of SIAs on routine health services in Pacific Island countries and territories—while SIA implementation led to benefits in some settings, it detracted from public health services in others [16].

Studies in this literature have used different methodologies, such as before-after comparisons and multivariate regression analyses [10,11,15]. Since the frequency of SIAs might be increased at times or in areas with poor routine services, there could be reverse causality in the SIA-routine vaccination relationship [13], which these methods would be unable to account for. In addition, given that exposure to SIAs is not random, another limitation of these methods is that they do not control for all factors that could shape exposure to SIA campaigns as well as routine immunization outcomes, and thus potentially bias results. For example, a country’s topography, level of development and health infrastructure could influence both the likelihood that a child visits routine health services and the decision to implement SIAs in the country. Alternatively, parents with a high level of trust in the national health system are more likely to allow their children to be immunized in SIA campaigns and to receive all the required vaccinations. In this article, we seek to account for the likely presence of such confounders by using the instrumental variable (IV) methodology, an approach that hasn’t been used in the literature investigating the impacts of SIAs.

## Methods

**Fig 1:**
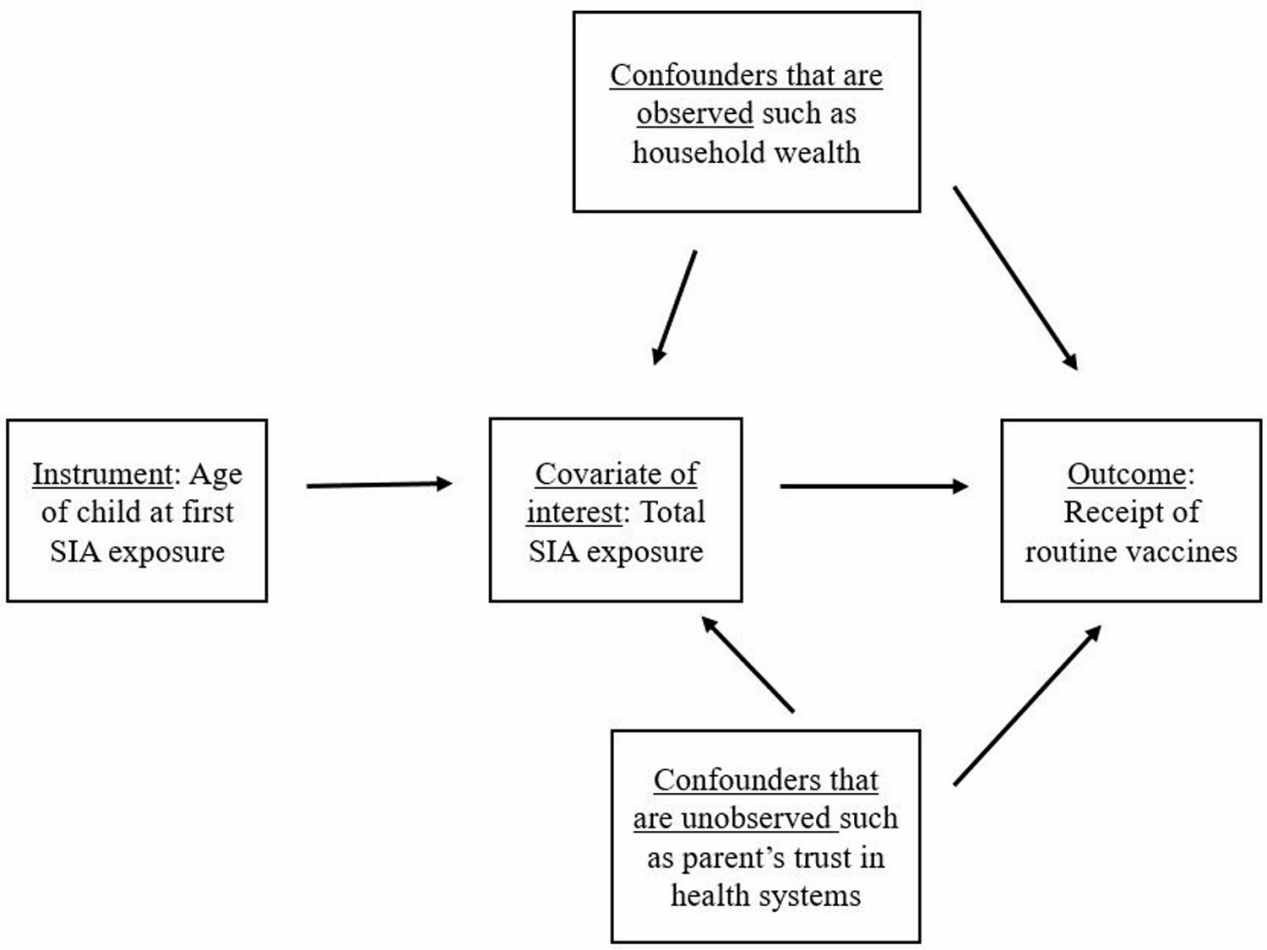
Schematic depiction of the use of an instrumental variable (IV) approach to identify the effect of SIA exposure on routine vaccination.

The outcome variable in this analysis is whether or not a child obtained key routine vaccines as reported in a household survey (which we describe below). We model it as a function of the total number of SIA campaigns a child could potentially have been exposed to and other control variables. Estimating the effects of total SIA exposure with an ordinary least squares approach might lead to biased results since unobservable factors such as a country’s level of development, health infrastructure and parents’ trust in the health system could shape both campaign exposure and the receipt of routine vaccines (as depicted in Fig 1). In order to deal with such potential sources of bias, we employ an IV approach—an econometric methodology that carves out exogenous variation in the variable of interest (which is uncorrelated with confounding variables) and uses this clean variation to identify the causal impacts of the instrumented covariate on outcomes. An IV analysis requires a variable (the instrument) that is strongly correlated with the potentially endogenous explanatory variable, but is unrelated to any of the unobserved factors that shape the outcome variable [17].

We use age at first SIA as an instrument for the total SIA exposure measure. Since most countries have ongoing SIA campaigns, it is likely that age at first SIA will predict total campaign exposure during childhood. We expect that the younger a child is at first SIA exposure, the more campaigns she is likely to have been exposed to in the period during which she needs to be immunized, leading to a negative relationship between the two variables. We hypothesize that the date of birth of any individual child is exogenous to the decision made by health planners on when to launch an SIA campaign and that couples do not time births according to SIA dates (perhaps so that their child can benefit as early as possible from SIA services). Thus, the age of children at first SIA depends on random variation in dates of birth and how old any given child is during her first campaign is unlikely to be associated with any potential sources of bias—note that in Fig 1 that there are no arrows flowing from any confounders towards the instrument. We can thus use the instrument to isolate a portion of total SIA exposure that is not shaped by other factors and identify unbiased effects on routine vaccination.

To conduct the IV approach, we first estimate the extent to which SIA exposure is explained by age at first exposure. We then use the predicted value of SIA exposure from this equation to estimate routine vaccination status ((see Appendix A in S1 File). The estimation process in both stages controls for other potential determinants of vaccination coverage—birth year indicators, birth in the rainy season, sex and birth order of child, mother’s educational level, rural residence, household electricity, and survey fixed effects [18–24].

Even though our main outcome of interest is dichotomous, we estimate linear probability models (LPM). We do so because the majority of the explanatory variables in the models are binary [17]. However, we also check the robustness of our results by estimating probit models. The standard errors we present are adjusted for clustering within survey-specific regions. We examine the results for each country separately since SIAs in different countries might have different implications. We use Stata 14 for the analysis.

## Data

Data for this study comes from two sources. We use WHO compiled data for all SIAs around the world, a dataset that includes the exact start date for all such campaigns between the years 1992 and 2013 (this data is obtained through UNICEF). We limit our sample to Bangladesh, Senegal, Togo, Gambia, and Cote d’Ivoire—countries in which a high proportion of SIAs are NIDs. We focus on NIDs only because of the following reasons. Firstly, we wish to capture the effect of campaigns that are conducted similarly across countries. NIDs meet this criterion since most countries follow WHO guidelines for these campaigns [25]. Secondly, since NIDs provide vaccines to all children in the targeted age group in a country and they take place over a short period of time (the WHO recommends that they be carried out over four to seven days or at most one month), it is possible to use dates of birth to identify children likely to have been exposed to these campaigns [25,26]. Finally, we exclude non-NID campaigns because it is often unclear what sub-national areas are targeted by these other campaigns. We define SIA exposure as the number of NIDs that occurred between a child’s date of birth and the time at which vaccination details were recorded (we use the exact start dates of all the NIDs for this exercise).

In order to identify the effect of SIAs on the utilization of routine vaccinations in the selected countries (those in which most campaigns are NIDs), we focus on time periods during which there are frequently occurring SIAs. The reason we impose this condition is that in several countries, there were some years during which SIAs were conducted at regular intervals, but some years during which they were not conducted at all. Including time periods with varying gaps between SIAs introduces the possibility of an inconsistently signed association between age at first SIA exposure and total SIA exposure. If SIAs were conducted at regular intervals, we would expect to find a linear relationship between these two variables—children first exposed to SIAs at an early age would have a higher total SIA exposure than children first exposed at a later age—and this would depend only on a child’s date of birth, which arguably has no systematic relationship with the timing of the first SIA after birth. If the duration between SIAs changes, the linear relationship between age at first SIA exposure and total SIA exposure disappears. For example, children who experience their first SIA early on in their lives but are exposed to no other or fewer subsequent SIAs due to a temporary halt on campaigns, might have lower total SIA exposure than children whose first SIA occurred at a later age but who faced periodically occurring SIAs after that. In order to have a valid IV, we omit time periods with irregular gaps between SIAs. Panel A in Table 1 lists the number of SIAs that occurred during the country-time periods covered in the study.

**Table 1:** Summary of data.

**Panel A: Sample overview**					
	Bangladesh	Senegal	Togo	Gambia	Cote d'Ivoire
Number of Supplementary Immunization Activities (SIAs)^1^	21	23	16	9	18
SIA years	2006–2013	2000–2005, 2010–2013	1996–1997, 2009–2013	2010–2012	2009–2012
Demographic and Health Surveys (DHS) years	2007, 2011, 2014	2005, 2010, 2012, 2014	1998, 2013	2013	2011
Cohorts included (Birth years)	2006–2013	2000–2005, 2010–2013	1996–1997, 2008–2013	2010–2012	2009–2011
Sample size of children	12,190	14,817	6,659	3,342	3,106
**Panel B: Summary statistics—means and standard deviations**^2^					
*Main variables*					
Exposure	5.228 (2.785)	4.505 (2.646)	4.769 (2.327)	3.762 (2.478)	9.856 (3.290)
Age at first SIA (in months)	4.720 (4.462)	5.144 (5.662)	4.750 (3.604)	1.664 (1.661)	1.599 (1.803)
Has DPT 3, Measles and BCG	0.866 (0.341)	0.736 (0.441)	0.663 (0.473)	0.854 (0.353)	0.541 (0.498)
*Control variables*^3^					
Rural residence	0.683 (0.465)	0.683 (0.465)	0.730 (0.444)	0.667 (0.471)	0.671 (0.470)
Birth order	2.392 (1.565)	3.766 (2.474)	3.675 (2.323)	3.670 (2.339)	3.569 (2.372)
Mother has primary education	0.300 (0.458)	0.199 (0.399)	0.326 (0.469)	0.147 (0.354)	0.223 (0.417)
Mother has secondary education	0.423 (0.494)	0.067 (0.250)	0.159 (0.366)	0.206 (0.404)	0.086 (0.280)
Mother has higher education	0.088 (0.283)	0.004 (0.065)	0.010 (0.100)	0.021 (0.144)	0.011 (0.103)
Household has electricity	0.601 (0.490)	0.392 (0.488)	0.278 (0.448)	0.285 (0.452)	0.461 (0.499)
Sex—female	0.487 (0.500)	0.493 (0.500)	0.501 (0.500)	0.494 (0.500)	0.507 (0.500)
Birth in rainy season	0.326 (0.469)	0.386 (0.487)	0.516 (0.500)	0.364 (0.481)	0.429 (0.495)

^1^The SIAs took place between the day of birth of the oldest child in the sample and the last DHS interview date. Campaigns are limited to national immunization days (NIDs).

^2^Standard deviations presented in parentheses below means.

^3^The regressions also control for birth year fixed effects.

Data on vaccination coverage and control variables is drawn from the standard Demographic and Health Surveys (DHS), which are nationally representative household surveys that are conducted in many low- and middle-income countries to collect data on key population, health and nutrition indicators. Previous studies like Bonu et al. (2003, 2004) and Haenssgen (2017) have probed the impact of SIA exposure on child-level outcomes with DHS or similar household survey data [10,11,15]. We use DHS data from the study countries and include children who were 12 to 59 months at the time of the DHS survey. It is recommended that required vaccinations be received during the first year of a child’s life and so by restricting the sample to children at least 12 months old, we should be able to capture receipt of these vaccines [27]. We set the upper age limit of the sample at 59 months because the DHS collects vaccination data for children born in the five years before a survey and the SIAs typically target children under five years of age [28]. Children in the study sample were born between 1996 and 2013 (Panel A, Table 1).

The WHO’s recommendation on infant immunizations calls for one dose of BCG, three doses of DTP, three doses of polio (either oral polio vaccines or inactivated polio vaccines), three hepatitis B, and one measles vaccine [29]. All countries included in this study adopted this recommendation. The outcome we examine in this analysis is a composite measure which accounts for whether a child has received all of the three following inoculations—DPT 3 (we focus on this vaccine in the DPT series since it provides immunity from the disease), measles, and BCG. We examine these vaccinations since they can be taken to indicate the reach of routine services through which these three vaccines are typically delivered [1]. We assume that a child has received a vaccine if her health card indicates so or if her mother reports that the child has obtained the vaccine.

We control for other potential determinants of vaccination coverage in the estimation models—birth year fixed effects (to account for any cohort-specific effects), sex of the child, birth order, the mother’s educational level, rural residence, and electricity (as a proxy for relative wealth). Children born in different seasons could be different since there is likely to be seasonal variation in birth rates as well as in death rates (for example, due to different early life exposures stemming from the seasonal change in disease incidence), and so we also include an indicator variable for children born during the rainy season in their country. Finally, we insert survey fixed effects to account for seasonal and reporting variation resulting from the time and other features of the survey through which data was collected. See Table A in S1 File for description of all variables used in the analysis.

## Results

Table 1 (Panel B) provides summary statistics for the variables used in this research. A typical child in Bangladesh, Senegal and Togo had been exposed to roughly five campaigns and first exposure in these countries was at about five months of age. Average SIA exposure in Gambia and Cote d’Ivoire was four and 10 respectively and mean age at first exposure in both countries occurred when children were about 1.5 months old. Children in Bangladesh were most likely to receive all of the three vaccines we examine—87 percent received DPT 3, measles, and the BCG vaccines. This figure was the lowest in Cote d’Ivoire—only 54 percent.

**Fig 2:**
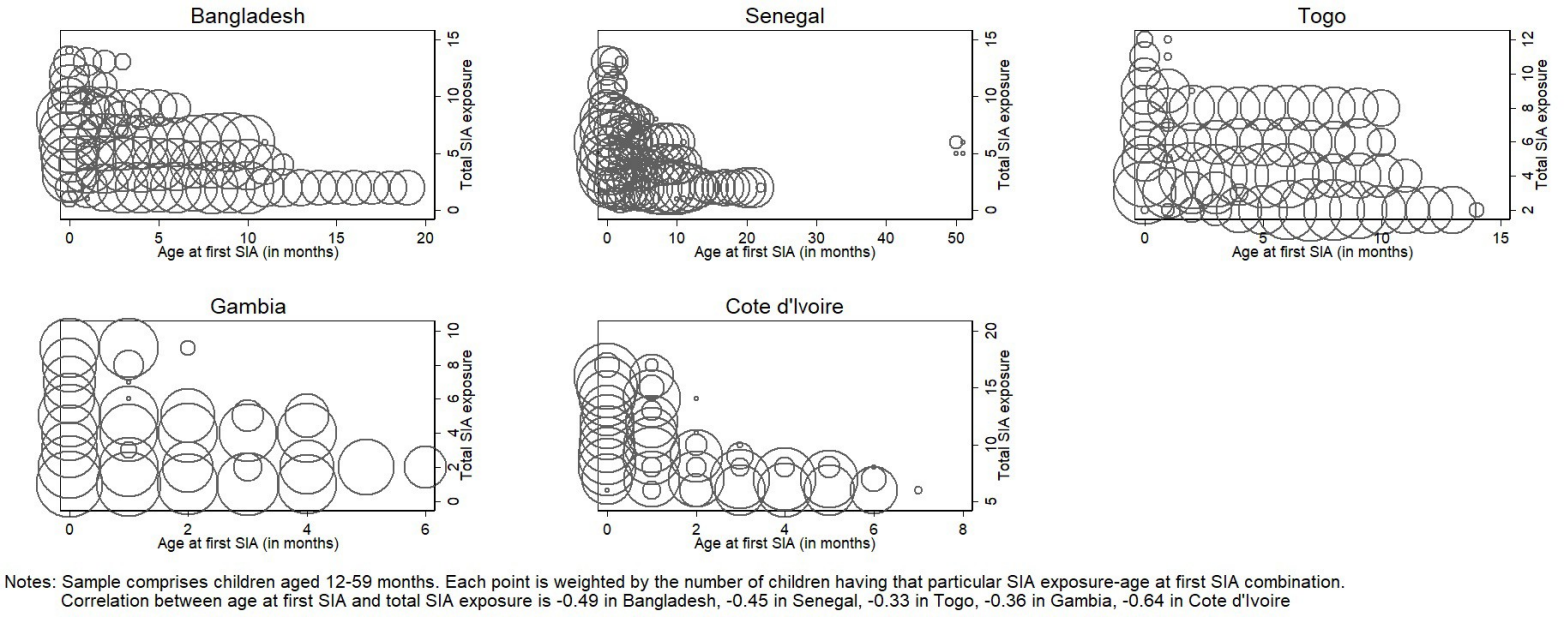
Impact of supplementary immunization activities (SIAs) on routine vaccinations—instrumental variable (IV) first stage.

Panel A of Table 2 contains the results of the first stage of the IV estimation which captures the relationship between age at first SIA (the instrument) and total potential SIA exposure. In all countries, a younger age at first SIA exposure allowed a child to be potentially targeted by more SIAs than if their first exposure was at an older age. The extent to which a one month increase in age at first SIA decreased total potential SIA exposure ranged from 0.037 in Bangladesh (column 1) to 0·447 in Gambia (column 4). All the coefficients on the instrument are significant at the one percent level and the F-statistics are sufficiently high, suggesting that we have a strong instrument [30,31]. The first stage results are also depicted in Fig 2.

**Table 2:** Impact of supplementary immunization activities (SIAs) on routine vaccinations—instrumental variable (IV) results.

	(1)	(2)	(3)	(4)	(5)
	Bangladesh	Senegal	Togo	Gambia	Cote d'Ivoire
**Panel A: FIRST STAGE**					
*Dependent variable*: *SIA exposure*					
Age at first campaign	-0.037***	-0.146***	-0.099***	-0.447***	-0.417***
	(0.001)	(0.005)	(0.002)	(0.010)	(0.014)
F-statistic of age at first campaign	54.95	86.86	63.46	718.39	1058.54
**Panel B: SECOND STAGE**					
*Dependent variable*: *Has DPT 3 + Measles + BCG*					
SIA exposure	-0.055**	-0.013**	-0.036**	-0.003	-0.006
	(0.022)	(0.006)	(0.015)	(0.008)	(0.011)
Mean SIA exposure in sample	5.228	4.505	4.769	3.762	9.856
Observations	12,190	14,817	6,659	3,342	3,106

Robust standard errors in parentheses.

*** p<0.01,

** p<0.05,

*

p<0.1. All models control for rural residence, birth order, mother's educational level (primary, secondary or higher), household electricity, sex (female), birth in rainy season, and fixed effects for survey round and birth year. See Table B in S1 File for full regression results (including control variables). The sample includes children aged 12–59 months. Standard errors are adjusted for clustering within survey-specific regions.

Table 2, Panel B, which presents the coefficients on the instrumented exposure variable, shows negatively signed impacts of SIA exposure in all the countries. The negative SIA effects on inoculations is not statistically significant in Gambia (column 4) and Cote d’Ivoire (column 5). Note though that the sample size for each of these countries is much smaller than for the other settings, which could have led to low statistical power. Bangladesh (column 1) experienced the largest impact—being exposed to an additional SIA in the country reduced the probability of having DPT3, Measles and BCG by almost 6 percentage points. The negative SIA coefficient in Senegal is of a magnitude of about one percentage point (column 2) and in Togo, it is almost four percentage points (column 3).

We present IV estimates using a probit model in Table 3. The magnitude and significance of the SIA exposure coefficients are almost identical to what we identified using an LPM model, thus pointing to the robustness of the results.

**Table 3:** Second stage instrumental variable (IV) results from probit models—marginal effects of supplementary immunization activities (SIAs) on routine vaccinations.

	(1)	(2)	(3)	(4)	(5)
	Bangladesh	Senegal	Togo	Gambia	Cote d'Ivoire
*Dependent variable*: *Has DPT 3 + Measles + BCG*					
SIA exposure	-0.055**	-0.013**	-0.039**	-0.002	-0.006
	(0.023)	(0.007)	(0.016)	(0.008)	(0.013)
Observations	12,190	14,817	6,659	3,342	3,106

Robust standard errors in parentheses.

*** p<0.01,

** p<0.05,

*

p<0.1. All models control for rural residence, birth order, mother's educational level (primary, secondary or higher), household electricity, sex (female), birth in rainy season, and fixed effects for survey round and birth year. The sample includes children aged 12–59 months. Standard errors are adjusted for clustering within survey-specific regions.

Table C in S1 File contains results for younger children aged 12–23 months since according to recommended vaccination schedules, children in this age group should have obtained the immunizations we examine [27]. In Table D in S1 File, the outcome is defined for children who had health cards with specific dates of receipt for the different vaccinations we examine, with the outcome coded one for those who obtained all three vaccines by age one and zero otherwise. Virtually all the SIA exposure coefficients in these two tables are negative. However, given that the sample restrictions lead to large drops in sample size, these regressions lack power.

In Table E in S1 File, we focus on the individual vaccines that are captured in the outcome variable we use for the main analysis—DPT 3 (panel A), measles (panel B) and BCG (panel C). The results in this table show negative SIA coefficients for almost all the individual routine vaccines. The broad consistencies in the direction of the effects point to the robustness of the main results of this analysis.

Finally, in two of the countries in our sample, we are able to incorporate some information on campaigns other than NIDs. Senegal had one mop-up activity and Cote d’Ivoire had one SNID for which coverage information is available. When we re-estimate results after adding these two vaccination efforts to the exposure count of children in the targeted areas (see results in Table F in S1 File), we find that the results persist in Senegal and become stronger in Cote d’Ivoire.

## Discussion

Through this investigation, we seek to contribute to the literature on whether SIAs have any effects on routine health service delivery. We employ the instrumental variable methodology for our analysis, an approach that has not been used in this literature before. We treat age of a child during the first SIA campaign after her birth as an instrument—we expect this variable to predict a child’s total SIA exposure through exposure to the first and subsequent campaigns. Furthermore, given that age at first campaign depends on the random variation in dates of birth, the variable is unlikely to be related to any unobserved factors that might systematically influence both SIA exposure and a child’s propensity to be vaccinated through routine health services. We are thus able to use the instrument of age at first SIA exposure to carve out exogenous variation in total SIA exposure and identify the unbiased effects of this covariate on the likelihood of obtaining the routine vaccinations DPT 3, measles and BCG. Our results show that children exposed to more campaigns had a lower likelihood of obtaining routine vaccines in all countries, but the coefficients fail to attain statistical significance in two of the settings. The point estimates that are significant indicate declines ranging from about one to six percentage points. The evidence we find is consistent with several other studies that have detected negative SIA impacts on routine vaccinations in certain settings [10,15].

Our analysis has some limitations. We restrict our study to countries in which most campaigns were national in scope since it is not always possible to identify the area of operation of other types of SIAs (such as child health days), and to link this information with data from DHS surveys. This sample restriction limits our focus to five countries and allows us to only identify the consequences of NIDs, an effect that might not capture the total impact of all SIAs on routine vaccine provision. The results we identify might apply only to the time period we examine (1996–2013) and stem from the way in which campaigns were implemented during these years. Due to data limitations, we are also unable to test for mechanisms through which the observed impacts could have materialized. Finally, we do not have information on actual exposure to SIAs and instead use potential exposure to SIAs. Since we employ an imperfect proxy for our covariate of interest, we have a case of error in the independent variable and so we likely obtain attenuated coefficients.

SIAs have been very successful in bringing about an increase in vaccination coverage, have allowed vaccines to be delivered during emergencies in countries like Afghanistan and Somalia, and have improved equity in immunization coverage [6,32,33]. However, the results of this research suggest that they can be disruptive to the delivery of routine services. If SIAs reduce access to routine vaccines, they will prevent the realization of full immunization rates. As of 2015, only about 60 percent of eligible children in low- and middle- income countries were completely immunized [34]. Maintaining uninterrupted routine health services during targeted campaigns is crucial since sustainable progress against vaccine-preventable diseases depends on the deployment of a wide range of inoculations, most of which take place through routine health systems [35]. We conclude by calling for greater support for routine services such that they are able to function seamlessly during supplementary health activities.

## Supporting information

S1 File Supporting information text and tables. (DOCX)

S2 File Study data. (DTA)

S3 File Analysis code. (DO)
