# Supplementary material for: The impact of supplementary immunization activities on routine vaccination coverage: An instrumental variable analysis in five low-income countries
Source: PLoS One. 2019 Feb 14;14(2):e0212049. doi: 10.1371/journal.pone.0212049 (PMC6375584; doi:10.1371/journal.pone.0212049)
Supplement: S1 File — (DOCX) [file pone.0212049.s001.docx]

**S1 File. Supporting information text and tables.**

**Appendix A: Instrumental variable (IV) analysis**

For the IV estimation, we use the following first stage equation:

E_i_ = α + γ*AGE_AT_FIRST_i_ + γ*Z_i_ + ε_i_  (1)

where E_i_ is the number of campaigns child i has been exposed to since birth, AGE_AT_FIRST_i_ is the child’s age in months at the time of the first SIA that occurs after the child’s birth, Z_i_ is a vector of control variables, and ε_i_ is the error term.

We then use the predicted value of SIA exposure from this equation to estimate routine vaccination status with the following second stage/outcome equation:

Y_i_ = α + β*Ehat_i_ + γ*Z_i_ + η_i_  (2)

where Y_i_ captures routine immunization, Ehat_i_ is the predicted value of the endogenous exposure variable generated after running (1), Z_i_ are the controls as above and η_i_ is the residual term.

**Table A: Definition of variables**

| *Variable* | *Definition* |
| --- | --- |
| SIA exposure | Total number of supplementary immunization activities (SIAs) a child could have been exposed to between birth and date of Demographic and Health Survey (DHS) interview |
| Age at first SIA | Age in months of child at first SIA after birth |
| Has DPT 3, Measles and BCG^1^ | 1 if the child received DPT 3, measles and BCG, zero otherwise |
| Rural residence | 1 if household lives in a rural area, 0 otherwise |
| Birth order | Order in which child was born to her mother |
| Mother has primary education | 1 if primary education is the highest education level attained, 0 otherwise |
| Mother has secondary education | 1 if secondary education is the highest education level attained, 0 otherwise |
| Mother has higher education | 1 if higher education is the highest education level attained, 0 otherwise |
| Household has electricity | 1 if child’s household has electricity, 0 otherwise |
| Sex – female | 1 if female, 0 if male |
| Birth in rainy season | 1 if child is born in the rainy season (based on information from http://sdwebx.worldbank.org/climateportal/), 0 otherwise |

Immunization data is recorded after either viewing health cards or, when the cards are not available, by asking mothers for this information. We consider having a vaccination date/mark on a child’s health card or a report by the mother as indication that the child has received the vaccination in question.

^1^This variable is defined for all children who have had at least one of these three vaccines. Note that we do not look at receipt of DPT 1 and DPT 2. We focus only on DPT 3 since as noted in the text in the article, this round in the DPT series provides immunity from the disease. Also it is likely that DPT 3 would be obtained only after a child received DPT 1 and DPT 2.

**Table B: Impact of supplementary immunization activities (SIAs) on routine vaccinations - Instrumental variable (IV) results**

|  | (1) | (2) | (3) | (4) | (5) |
| --- | --- | --- | --- | --- | --- |
|  | Bangladesh | Senegal | Togo | Gambia | Cote d'Ivoire |
| **FINAL OUTCOME MODEL/SECOND STAGE** | | |  |  |  |
| *Dependent variable: Has DPT 3 + Measles + BCG* | | |  |  |  |
| SIA exposure | -0.055** | -0.013** | -0.036** | -0.003 | -0.006 |
|  | (0.022) | (0.006) | (0.015) | (0.008) | (0.011) |
| Rural residence | 0.012 | 0.026** | 0.059 | 0.108*** | -0.075*** |
|  | (0.008) | (0.013) | (0.055) | (0.035) | (0.024) |
| Birth order | -0.009*** | -0.003* | -0.002 | 0.005 | -0.001 |
|  | (0.003) | (0.002) | (0.003) | (0.004) | (0.005) |
| Mother has primary education | 0.068*** | 0.091*** | 0.060* | -0.025*** | 0.140*** |
|  | (0.013) | (0.015) | (0.031) | (0.007) | (0.018) |
| Mother has secondary education | 0.129*** | 0.146*** | 0.139*** | 0.004 | 0.207*** |
|  | (0.017) | (0.013) | (0.029) | (0.017) | (0.037) |
| Mother has higher education | 0.172*** | 0.212*** | 0.201*** | -0.082 | 0.353*** |
|  | (0.019) | (0.031) | (0.043) | (0.058) | (0.037) |
| Household has electricity | 0.046*** | 0.074*** | 0.087*** | 0.006 | 0.101*** |
|  | (0.013) | (0.014) | (0.026) | (0.019) | (0.034) |
| Sex - female | -0.015** | -0.005 | -0.013 | -0.016 | -0.024* |
|  | (0.007) | (0.006) | (0.013) | (0.010) | (0.014) |
| Birth in rainy season | -0.022*** | -0.016** | -0.029** | -0.012 | 0.022 |
|  | (0.007) | (0.007) | (0.012) | (0.017) | (0.024) |
|  |  |  |  |  |  |
| Mean SIA exposure in sample | 5.228 | 4.505 | 4.769 | 3.762 | 9.856 |
| Observations | 12,190 | 14,817 | 6,659 | 3,342 | 3,106 |

Robust standard errors in parentheses. *** p<0.01, ** p<0.05, * p<0.1. All models control for rural residence, birth order, mother's educational level (primary, secondary or higher), household electricity, sex (female), birth in rainy season, and fixed effects for survey round and birth year. The sample includes children aged 12-59 months. Standard errors are adjusted for clustering within survey-specific regions.

**Table C: Impact of supplementary immunization activities (SIAs) on routine vaccinations - Instrumental variable (IV) results, Children 12-23 months**

|  | (1) | (2) | (3) | (4) | (5) |
| --- | --- | --- | --- | --- | --- |
|  | Bangladesh | Senegal | Togo | Gambia | Cote d'Ivoire |
| **FINAL OUTCOME MODEL/SECOND STAGE** | | |  |  |  |
| *Dependent variable: Has DPT 3 + Measles + BCG* | | |  |  |  |
| SIA exposure | -0.094* | -0.091 | -0.058 | -0.104 | 0.017 |
|  | (0.052) | (0.065) | (0.066) | (0.080) | (0.025) |
|  |  |  |  |  |  |
| Mean SIA exposure in sample | 2.971 | 2.328 | 2.706 | 1.539 | 6.897 |
| Observations | 3,235 | 4,889 | 2,567 | 1,473 | 1,385 |

Robust standard errors in parentheses. *** p<0.01, ** p<0.05, * p<0.1. All models control for rural residence, birth order, mother's educational level (primary, secondary or higher), household electricity, sex (female), birth in rainy season, and fixed effects for survey round and birth year. The sample includes children aged 12-23 months. Standard errors are adjusted for clustering within survey-specific regions.

**Table D: Impact of supplementary immunization activities (SIAs) on routine vaccinations - Instrumental variable (IV) results, Vaccines obtained within first year of life**

|  | (1) | (2) | (3) | (4) | (5) |
| --- | --- | --- | --- | --- | --- |
|  | Bangladesh | Senegal | Togo | Gambia | Cote d'Ivoire |
| **FINAL OUTCOME MODEL/SECOND STAGE** | |  |  |  |  |
| *Dependent variable: Has obtained DPT 3 + Measles + BCG in first year of life* | | | | | |
| SIA exposure at age one | -0.006 | -0.026 | -0.017 | -0.027 | -0.178 |
|  | (0.272) | (0.046) | (0.038) | (0.019) | (0.430) |
|  |  |  |  |  |  |
| Mean SIA exposure by age one in sample | 2.343 | 2.473 | 1.985 | 2.578 | 4.527 |
| Observations | 7,111 | 7,181 | 3,640 | 2,784 | 2,007 |

Robust standard errors in parentheses. *** p<0.01, ** p<0.05, * p<0.1. All models control for rural residence, birth order, mother's educational level (primary, secondary or higher), household electricity, sex (female), birth in rainy season, and fixed effects for survey round and birth year. The outcome variable is defined for children between the ages of 12 and 59 months who have health cards that contain vaccination dates. Standard errors are adjusted for clustering within survey-specific regions.

**Table E: Impact of supplementary immunization activities (SIAs) on routine vaccinations - Final outcome model of instrumental variable (IV) specification for individual vaccines**

|  | (1) | (2) | (3) | (4) | (5) |
| --- | --- | --- | --- | --- | --- |
|  | Bangladesh | Senegal | Togo | Gambia | Cote d'Ivoire |
| **Panel A** |  |  |  |  |  |
| *Dependent variable: Has DPT 3* | |  |  |  |  |
| SIA exposure | -0.015 | -0.006 | -0.049** | -0.005 | -0.010 |
|  | (0.012) | (0.005) | (0.021) | (0.005) | (0.016) |
|  |  |  |  |  |  |
| Mean SIA exposure in sample | 5.227 | 4.505 | 4.768 | 3.762 | 9.847 |
| Observations | 12,178 | 14,767 | 6,636 | 3,334 | 3,076 |
| **Panel B** |  |  |  |  |  |
| *Dependent variable: Has Measles* | |  |  |  |  |
| SIA exposure | -0.048** | -0.019*** | -0.029* | -0.007 | 0.009 |
|  | (0.022) | (0.005) | (0.017) | (0.005) | (0.012) |
|  |  |  |  |  |  |
| Mean SIA exposure in sample | 5.227 | 4.505 | 4.768 | 3.762 | 9.848 |
| Observations | 12,148 | 14,698 | 6,598 | 3,341 | 3,071 |
| **Panel C** |  |  |  |  |  |
| *Dependent variable: Has BCG* | |  |  |  |  |
| SIA exposure | 0.005 | -0.004 | -0.013 | -0.003* | -0.014 |
|  | (0.011) | (0.003) | (0.011) | (0.002) | (0.011) |
|  |  |  |  |  |  |
| Mean SIA exposure in sample | 5.227 | 4.505 | 4.768 | 3.762 | 9.854 |
| Observations | 12,178 | 14,809 | 6,655 | 3,342 | 3,095 |

Robust standard errors in parentheses. *** p<0.01, ** p<0.05, * p<0.1. All models control for rural residence, birth order, mother's educational level (primary, secondary or higher), household electricity, sex (female), birth in rainy season, and fixed effects for survey round and birth year. The sample includes children aged 12-59 months. Standard errors are adjusted for clustering within survey-specific regions.

**Table F: Impact of supplementary immunization Activities (SIAs) on routine vaccinations - Instrumental variable (IV) results, Adding information on all campaigns with coverage data**

|  | (1) | (2) |
| --- | --- | --- |
|  | Senegal | Cote d'Ivoire |
| **FINAL OUTCOME MODEL/SECOND STAGE** | |  |
| *Dependent variable: Has DPT 3 + Measles + BCG* | | |
| SIA exposure | -0.013** | -0.023** |
|  | (0.006) | (0.011) |
|  |  |  |
| Mean SIA exposure in sample | 4.511 | 10.129 |
| Observations | 14,817 | 3,106 |

Robust standard errors in parentheses. *** p<0.01, ** p<0.05, * p<0.1. All models control for rural residence, birth order, mother's educational level (primary, secondary or higher), household electricity, sex (female), birth in rainy season, and fixed effects for survey round and birth year. The sample includes children aged 12-23 months. Standard errors are adjusted for clustering within survey-specific regions.
